# Brain-inspired model for early vocal learning and correspondence matching using free-energy optimization

**DOI:** 10.1371/journal.pcbi.1008566

**Published:** 2021-02-18

**Authors:** Alexandre Pitti, Mathias Quoy, Sofiane Boucenna, Catherine Lavandier

**Affiliations:** Laboratoire ETIS, CY Cergy Paris University, ENSEA, CNRS, UMR8051, Cergy, France; University of California at Berkeley, UNITED STATES

## Abstract

We propose a developmental model inspired by the cortico-basal system (CX-BG) for vocal learning in babies and for solving the correspondence mismatch problem they face when they hear unfamiliar voices, with different tones and pitches. This model is based on the neural architecture INFERNO standing for Iterative Free-Energy Optimization of Recurrent Neural Networks. Free-energy minimization is used for rapidly exploring, selecting and learning the optimal choices of actions to perform (eg sound production) in order to reproduce and control as accurately as possible the spike trains representing desired perceptions (eg sound categories). We detail in this paper the CX-BG system responsible for linking causally the sound and motor primitives at the order of a few milliseconds. Two experiments performed with a small and a large audio database show the capabilities of exploration, generalization and robustness to noise of our neural architecture in retrieving audio primitives during vocal learning and during acoustic matching with unheared voices (different genders and tones).

## Author summary

We designed a developmental architecture inspired by the cortico-basal system for early vocal learning. Our neural system explores, evaluates and strengthens the motor primitives that match the best the sound repertoire created also dynamically. After a babbling process in which the network tests and aligns pronounced sound and motor vocal tracks, it is used for listening to novel voices, solving the correspondence problem.

## Introduction

Infants learn language by matching perceptually the low-level auditory features they hear with the articulatory motions they perform for vocal production. Perceptual ambiguity or mismatch occurs when they have to interpret someone else’s speech based on their own sound repertoire, which is akin to the correspondence problem [1].

In order to interpret correctly which sound has been pronounced and which articulatory motion is producing it, brain networks have to be organized flexibly early in infancy, for retrieving and categorizing memory sequences of orders of milliseconds [2, 3].

We propose a brain-inspired model for the early vocal learning and the emergence of sound categorization performed during infancy. So far, few computational models of language processing exist and fewer are brain-inspired [4–8]. In this introduction, we will first review computational models of early vocal learning. In the second, we will present our architecture and discuss the advantages and limitations in comparison to these models.

### Review of computational models of early vocal learning

Recent computational models of vocal production have been reviewed by Warlaumont and Finnegan [9]. As they state in their paper, many computational models of vocal learning focus on the production of a fixed vowel repertoire only [10]. In other models, it is the speech production that is already organized syllabically, which includes static categorizations of vowels learned [11–13]. These models do not address the question of how repertoires of consonant syllables can be constructed, or of how more complex chunks can be created. In the majority, the problems addressed involve the acoustic matching of static categorizations, which does not account for variability in timing integration, switching between self-learning and interaction with a caregiver, noise and errors in the perceptual categorization of unfamiliar voices or in other languages.

Nevertheless, some address this issue: for instance, Miura’s study shows a robot that is capable of mutual imitation for vowel learning during human-robot interaction and improves vowel recognition and imitation [14]. In this study, the robot has lips to limit its exploration space and to improve its articulatory imitation. The self-mirroring plays an important role to guide the robot to obtain clearer vowel prototypes through the ability to self-hear and self-correct [15].

In one of our recent works, Dermy, Valentin and colleagues present a sensory-motor architecture based on a neural network allowing a robot to recognize vowels in a multi-modal way as a result of human mimicking [16, 17]. The robot learns online to associate what it is doing with what it is seeing and hearing. In earlier works, Oudeyer studied how robots can develop and build a discrete speech code without linguistic knowledge [18]. These studies underline the issue of correspondence problem where the robot learns to vocalize by interacting with a robot or human partner [19]. However, the sound characteristics used are mostly the first two formants to distinguish vowels and the repertoire of sound categories is mostly limited to few vowel prototypes and syllables.

Besides, several interesting models have been considered where the importance of timing, self-supervised learning and continuous vocal imitation through interaction with a caregiver are discussed. In these models, recurrent neural networks, and spiking neural networks have been proposed with reward modulation for learning audio-motor spatio-temporal patterns [11, 20, 21].

For instance, Warlaumont and colleagues [9] have exploited spiking recurrent neural networks using Spike Timing-Dependent Plasticity (STDP) for synchronizing contingent neurons between audio and motor maps for learning a repertoire of syllables. Spatio-temporal clusters are learned in an unsupervised manner and the global network self-organizes into a reservoir of audio primitives. Similar research has been done by Kanda and colleagues [11] and by Kroeger and colleagues [8, 22].

The advantages of spiking networks consist in detecting precise delays across signals, anticipating several dynamics in parallel, and dynamically switching the direction of the control flow from perception-driven control (external influence on internal dynamics) to motor-driven control (internal influence on external dynamics). Some disadvantages lie on the level of noise and the variability of the input dynamics in spiking recurrent neural networks, making the learning and control of a large number of clusters difficult, in comparison to supervised learning methods. This kind of spiking recurrent networks have been already evaluated in similar research on self-perception in visuo-motor control [23], and on visuo-tactile integration [24, 25].

Similar issues occur in recurrent neural networks or in reservoir computing with the so-called vanishing gradient problem, which corresponds to the temporal window which the RNNs can accurately predict [6, 11, 26, 27].

Another interesting framework based on intrinsic motivation has been investigated by Moulin-Frier and colleagues [18, 28] and by [22] to explore a repertoire of articulatory motions and syllables. The intrinsic motivation mechanism combines reinforcement learning and novelty detection to optimize the learning curve by discretizing the parameter space in small portions [29, 30]. This learning paradigm follows a developmental stage, which makes it possible an autonomous exploration and a gradual discovery of the agent’s own motor repertoire. Although the motor space is high in their study (29 articulations), the sensory space is small in comparison as it is constituted of three frequencies to track. Also, it is not clear how the model can expand to higher dimensions and to more complex chunks since the convergence time can be high. Furthermore, their architecture is modeled at a high level of abstraction and does not specifically reproduce neural mechanisms or brain architecture at different time-scales.

### Our proposal for early vocal learning

In our model, we try to keep advantages of the presented models, but go further against their drawbacks. For instance, we put forward ideas of predictive coding [31], and free-energy minimization as in Friston [32], along with the unsupervised learning mechanism of STDP to propose a neural architecture that discovers and learns by trials and errors the motor patterns associated with the relevant sound patterns.

The Free-energy minimization principle introduced by Friston [32–34] instantiates that surprise, or error prediction, can be minimized through an active inference process or a control problem. The variational free-energy on dynamics (*effect*) can be optimized by neural control or action (*cause*). Learning the relationship between cause and effect permits to anticipate errors and to correct the system’s response even in presence of novel input, which differs from the classic reinforcement learning paradigm. On a memory recall problem, long-range memory sequences can be dynamically controlled and actively retrieved as attractors. On a sensorimotor problem, free-energy minimization is used for rapidly exploring, selecting and learning the optimal choices of actions to perform (eg sound production) in order to reproduce and control the most accurately as possible the spike trains representing desired perceptions (eg sound categories).

We hypothesize that free-energy minimization will permit the control of the dynamics of spiking neural networks and the learning of a large repertoire of audio chunks in comparison to the other models found in the literature. Of particular importance, the free-energy optimization should permit the rapid exploration and convergence of the model during the learning stage even with the presence of noise, and should permit also to infer categories even in the presence of novel input. Free-energy minimization does not require the gradual freezing of the parameters’ space as found in intrinsic motivated models, which constraints the learning stage into discrete and longer periods.

We propose to model a neural architecture inspired by the cortico-basal circuits responsible for processing and organizing the learning between the audio sensory map and the motor control; see [4, 35].

In the first experiment, we will present results on vocal learning from one speaker only (e.g., one-to-one correspondence) and how our architecture rapidly constructs its sound repertoire by free-energy minimization of internal signals. In the second experiment, we will show how this architecture solves the correspondence problem from six different speakers (e.g., one-to-many correspondence), learning a model from the six speakers. We show in that experiment how the predictive coding architecture can help to be robust to noise, for inference.

In comparison to [36], we use the same neural architecture althought the difference lies on the task applied on vocal learning and on the size of the audio dataset constituted of more than ten thousand audio (MFCC) vectors, which is higher than the 25 vectors only in our previous study. We prove therefore the scalability of our network to problems of higher dimensions.

Although several reward modulated spiking recurrent neural networks exist on vocal learning [9, 37], to our knowledge, no one has achieved such performances: (1) for constructing a large audio repertoire and (2) being robust to noise during acoustic matching even from different speakers.

The paper is organized as follows. In section, we will describe our model and present some neural justifications supporting it. In section, we will present the neural architecture and its learning mechanisms. In section, we will present the two experimental setups for vocal learning and acoustic matching, respectively from a limited learning database (only one speaker, 3 minutes length) and from a larger database (six speakers of different genders, 27 minutes length). The results of these two experiments are set out and discussed in section.

## Proposal framework for feature extraction and sequence learning

### State of the art and model justification

We propose a neural architecture that models broadly the interaction between the cortical layers (CX) and the Basal Ganglia (BG) for retrieving sound units. The working memory is developed within the same framework of Free-Energy [34, 38, 39] that combines predictive coding and reinforcement learning to code information and to minimize online error by exploiting noise.

Our architecture uses the rank-order algorithm to model spiking neural networks (SNN) [40]. This algorithm models the temporal order between neurons and permit to simulate well the mechanism of Spike Timing-Dependent Plasticity (STDP) [41–43] in order to learn temporal delays between pre- and post-synaptic firing neurons. We also exploit reinforcement learning and intrinsic noise in order to realize a stochastic descent gradient and novelty detection in line with the framework of free-energy minimization [33].

We propose that these different mechanisms serve for the learning of temporal delays between neurons in a self-organizing manner and makes possible the discovery of causes and effects necessary for active inference and predictive coding. This work extends previous research in which we developed several models of Working Memory (WMs) using SNNs corresponding to different brain areas. For example, our previous models exploited noise and novelty detection to iteratively infer a model and minimize error prediction, either to control one system’s dynamics in model-free networks of the hippocampus [44] and of the basal ganglia [36], or to select dynamically a better controller in a model-based network of the prefrontal cortex [45].

In [36], we modeled a compound network constituted of a cortical system based on unsupervised learning and a basal ganglia system based on reinforcement learning to control long-range memory sequences of spikes –, above 1000 iterations without loss,– and to solve the so-called temporal credit assignment problem by inferring causes and effects, even with long-range delays. Because of its ability to optimize and control dynamics iteratively using prediction error, known as free-energy minimization, we named our network INFERNO, standing for Iterative Free-Energy Optimization for Recurrent Neural Networks [36]. Our original paper [36] extensively analyzes with different parameters and metric the performances of the INFERNO architecture used in this paper. In particular, we show how the robustness to delays arises from the free-energy minimization enabling the control of the input to the recurrent network. Related work is currently performed for the learning and chaining of sound primitives [46] and motor primitives [47].

Presently, we apply the INFERNO network to speech learning (perception and production) for the recognition and generation of audio memory sequences.

**Fig 1:**
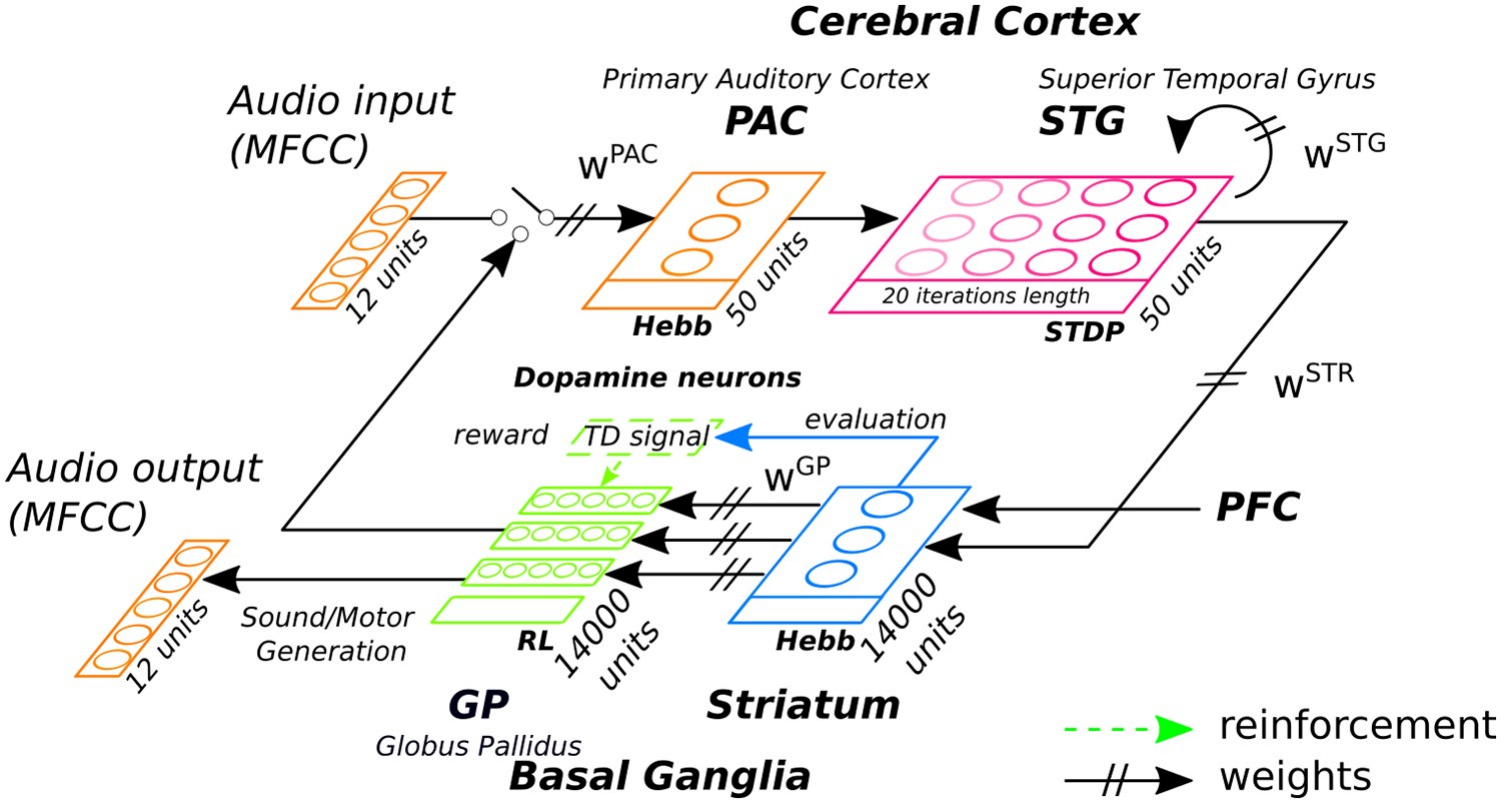
Framework of the INFERNO architecture for audio primitive retrieving based on iterative optimization through the cortico-basal ganglia loop (CX-BG). The Primary Auditory Cortex (PAC) receives and categorizes the audio vectors as a first stage, the Superior Temporal Gyrus cortex (STG) integrates over time its outputs that are eventually categorized by the Striatum (STR) in the basal ganglia. The Globus Pallidus (GP) searches and retrieves the audio vectors that best match the STG dynamics recognized by the striatal units. The iterative optimization process is carried out by minimizing noise with a temporal difference reinforcement signal.

In this framework, we can apprehend the cortico-striatal loop as two learning systems that attempts to perform an optimal control and resolve error prediction among their dynamics. In Fig 1, we display our framework with the cortical system (CX) composed of the Primary Auditory Cortex (PAC) system and the Superior Temporal Gyrus (STG) layer modeled with SNNs to encode incoming inputs, the Striatum layer (STR) that categorizes the state of the STG dynamics and the Globus Pallidus (GP) that attempt to retroactively control the input dynamics of the PAC and STG with a reentrant loop. The error prediction is evaluated and minimized over time by supervision of the STR units (the critic) and by noise generation and stochastic exploration performed on the GP output layer (the actor).

### Neural foundations for error-minimization in the cortico-striatal systems

In different brain areas, working memories (WMs) are hypothesized as embedding neural processes with forward and inverse models that can encode, anticipate and eventually control incoming signals to be more robust and to overcome their variability [48–50]. Two brain areas namely the Basal Ganglia (BG) that selects actions with respect to current states [51] and the Prefrontal Cortex (PFC) that represents forthcoming actions with respect to current contexts [3, 52, 53], are important for embedding these WMs; see Fig 1. Being part of two different loops but connected at the BG level, they realize reactive (BG) and proactive (PFC) control, processing information differently and at different speed.

On the one hand, some evidence indicates that the striatum in BG has a principal function in learning-related plasticity associated with selecting one set of actions from many, resulting in the acquisition of habitual behavior [54, 55]. On the other hand, PFC achieves behavioral planning in terms of the end result, rather than in terms of the movement required to perform the task [56, 57].

Graybiel and Grafton suggest in [58] that proactive control is associated with sustained and/or anticipatory activation of lateral PFC, which reflects the active maintenance of task goals. By contrast, reactive control should be reflected in transient activation, along with a wider network of additional brain regions such as the BG. Therefore, these two control mechanisms differ in terms of their involvement during learning and retrieving tasks or sequences, with the BG dynamics working at a faster pace than the PFC.

In the computational neurosciences domain, reactive and proactive control relate to what is called model-free and model-based systems in Reinforcement Learning (RL) [51, 59–61], having one system for stimulus-response tasks performing greedy-like optimization –, which means sensorimotor RL tasks (e.g., motor exploration and sound matching),– and the other learning distinct policies for prediction –, which serves for planning goal-directed behaviors (e.g., chunking syllabes into words). Koechlin and colleagues explain how these two systems contribute to adaptive behavior [53] and to language processing [62].

These two features of planning and optimization are also linked to what is now called the Bayesian theory of the brain [63, 64] and to the paradigm of predictive coding for cognition [31, 33, 38]. These general theories describe how our expectations (as well as our errors) on sensory inputs are used as attention signals to adjust the prior expectations for the next events. Brain areas are hypothesized as using error prediction as a core information to mutually *control* their dynamics, not just to bind them together.

Under this framework, two or more brain networks can interact dynamically (e.g., the Cortex CX with the Basal Ganglia BG) so that we have always one network (e.g., the controller) that infers the reliability of another (e.g., the observer) with respect to a specific context. Along with Bayes theory, predictive coding also has a link with optimal control theory [65], which we think interesting in terms of perspectives for modeling the corticostriatal system as it turns the problem of learning and retrieving memory sequences into a control problem.

This neural process has been particularly studied for speech and language sequences because auditory modality is the sense that is especially sensitive to temporal structure. In the case of speech production, Romanski and colleagues propose that the phonotopical level requires the implementation of high-order models for encoding words or sentences as articulatory vocal tracks [66].

## Materials and methods

We here present the neural architecture INFERNO used for predictive coding associated with CX and BG. We then describe the coding mechanism used for modeling the spiking neurons and the learning mechanisms associated with temporal order and rank coding. We then define the experimental setup and the parameters used in the context of audio primitive retrieval for encoding the audio signals.

### The recurrent network INFERNO

**Fig 2:**
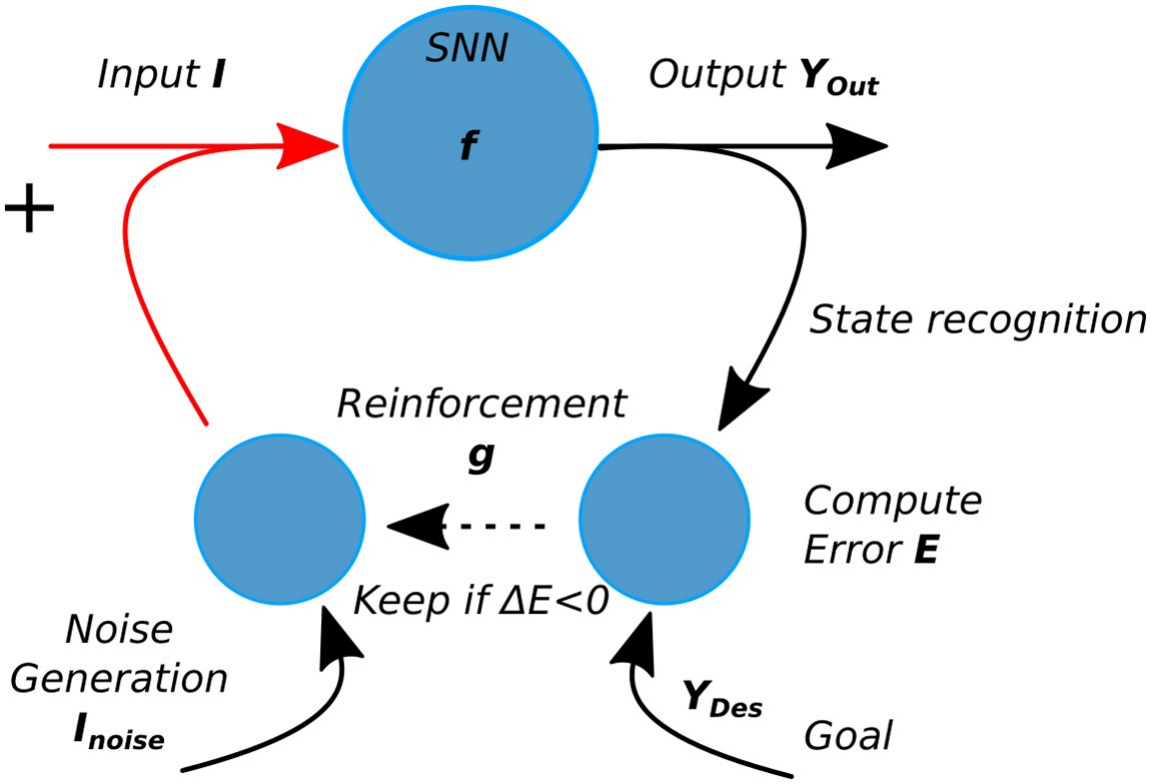
Stochastic descent gradient optimization used to control the neural dynamics. Free-energy (noise) is injected as Input in the network. After a period of time, the Output vector is read to recognize the state and its value is compared to a goal vector. If the variational error E is decreasing, the stochastic descent gradient keeps the current Input. After several cycles, the Input converges to its optimal values that minimizes error and maximizes the state recognition stage.

The neural architecture INFERNO [36] consists of two coupled learning systems arranged as in Fig 2. The first network corresponds to one recurrent neural network of spiking neurons (SNNs) and the second network consists of one associative map. The SNN implements a forward model of the incoming signals whereas the associative map implements an inverse model aimed at retrieving and controlling those signals. The inverse-forward controller can be modeled with the function *Y*_*out*_ = *f*(*I*) for the SNN and with the function *I* = *g*(*Y*_*out*_) for the associative map, in which *I* is the input vector and *Y*_*out*_ are the output dynamics.

In order to minimize error, the second network generates intrinsic noise *I*_*noise*_ to control the dynamics of the first, following a RL mechanism. The activity of the SNN *Y*_*out*_ is compared to one desired goal vector *Y*_*des*_ to compute the error *E* between *Y*_*des*_ and *Y*_*out*_ and the current input is kept for the next step *I*(*t*+ 1) = *I*(*t*)+ *I*_*noise*_, if and only if it diminishes the gradient *ΔE*. Over time, *I* converges to *I*_*opt*_ its optimum value, and *Y*_*out*_ converges to *Y*_*des*_, the desired vector. This scheme is in line with actor-critic algorithms and predictive coding. Its organization is similar to novel architectures combining two or more competitive neural networks such as auto-encoders or generative adversarial networks.

We showed in [36] that this variational process is similar to a stochastic descent gradient algorithm performed iteratively and can solve the temporal credit assignment problem for delays above tens of iterations. For instance, the convergence to the desired goal after a certain delay can be viewed as the retrieval of a memory sequence for such duration. Furthermore, the free-energy minimization is generative in the sense that it can retrieve novel solutions *I* for the same output *Y*. This can be viewed as a synchronization process toward attractor memories [67].

### Neuron model—Rank-Order Coding algorithm

We use the rank-order coding (ROC) algorithm to model integrate-and-fire neurons and the STDP rule [40, 68]. In their study, Laurent Perrinet and Simon Thorpe showed that rank-order codes model well the STDP in discrete time steps. We observed also such behaviors in recurrent networks in [36, 69] and our results showed that it is possible to use it effectively to approximate the integrate-and-fire type of neurons and temporal dependencies. Other models can be applied but the computation of the rank-order neurons is fast and easy to implement, which is convenient for our study and for future implementation in robots for online processing.

**Fig 3:**
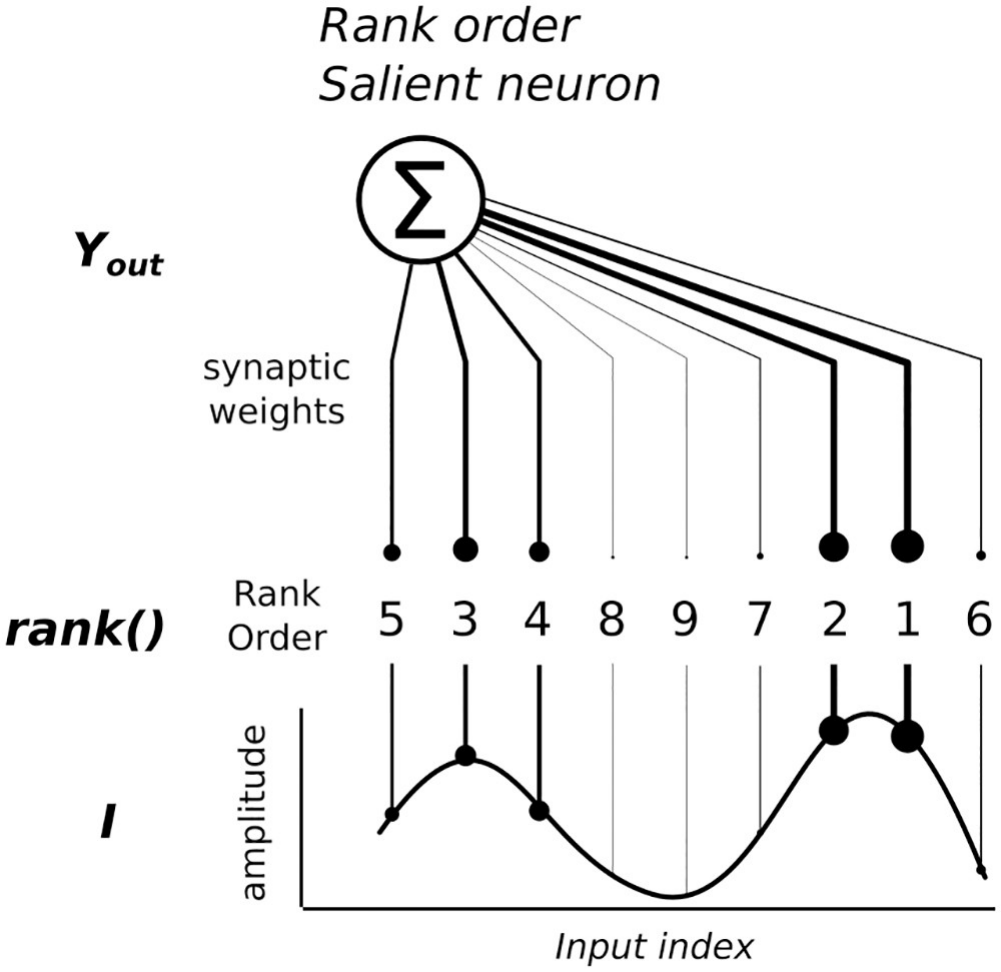
Rank-Order Coding principle [68]. This type of neuron encodes the rank code of an input signal. Its amplitude is translated into an ordered sequence and the neuron’s synaptic weights are associated with this sequence. In our example, the neural activity is salient to this particular order, which is seen in the line widths of the synaptic weights.

For instance, ROC neurons can translate ordered spatio-temporal patterns into ranked weights, see Fig 3. The more similar the sequence order of the incoming signals, the higher the amplitude level of the ROC neurons. Conversely, the less similar the sequence order of the incoming signals, the lower the amplitude level of the ROC neurons.

If we adopt an ordinal ranking sensitive to the amplitude level of incoming units as displayed in Fig 3, this coding strategy adequately retranscribes the Hebbian rule of “neurons that fire together wire together”. These units can model accurately the properties of common neural populations in the neocortex.

$$\begin{array}{c} Y_{i}^{STG}\left(t\right)=Y_{i}^{PAC}\left(t\right)+\sum_{j=1}^{50}\sum_{k=1}^{20}w_{jk}^{STG}rank\left(Y_{k}^{STG}\left(t-1\right)\right) \end{array}$$ (1)

where the sum over ‘*k* implements a 20 iterations window buffer.

The equations of the rank-order coding algorithm that we used are as follows. The neurons’ output *Y* is computed by processing the dot product between the function *rank()* sensitive to a specific rank ordering within the input signal vector *I* and the synaptic weights *w*; *w* ∈ [0, 1]. As an example, one possible rank function can be $rank\left(i\right)=\frac{1}{1+i}$ that decreases monotonically with respect to the *i*^*th*^ rank of one item. For the PAC network, we have for an input vector signal *X* of dimension *M* = 12 and for a population of *N* = 50 neurons (*M* afferent synapses):

$$\begin{array}{c} Y_{n}^{PAC}=\sum_{m}^{M}rank\left(X\right)\;w_{nm}^{PAC},\forall n\in N \end{array}$$ (2)

For the STR network, we have for a vector signal of dimension *M* = 50 and for a population of *N* = 14000 neurons (*M* afferent synapses):

$$\begin{array}{c} Y_{n}^{STR}=\sum_{m}^{M}rank\left(Y_{m}^{STG}\right)\;w_{nm}^{STR},\forall n\in N \end{array}$$ (3)

The rank function *rank()* can be implemented classically as a power law of the argsort() function normalized between [0, 1] for modeling the STDP. This warranties that the density distribution is bounded and that the weight matrix is sparse, which makes the rank-order coding neurons similar to radial basis functions. This attribute permits them to be used as receptive fields so that the more distant the input signal is to the receptive field, the lower is its activity level. The updating rule of the weights is similar to the winner-takes-all strategy in Kohonen networks [70] with an adaptive learning rate *α*_*n*_, ∀*n* ∈ *N*. For the best neuron *Y*_*b*_, we have for STR network:

$$\begin{array}{ccc} \Delta w_{bm}^{STR} & = & \alpha_{b}\left(rank\left(Y_{m}^{STG}\right)-w_{bm}^{STR}\right),\forall m\in M \end{array}$$ (4)

The same updating rule applies for the PAC and STG networks.

$$\begin{array}{ccc} \Delta w_{bm}^{PAC} & = & \alpha_{b}\left(rank\left(X\right)-w_{bm}^{PAC}\right),\forall m\in M \end{array}$$ (5)

$$\begin{array}{ccc} \Delta w_{bm}^{STG} & = & \alpha_{b}\left(rank\left(Y_{m}^{PAC}\right)-w_{bm}^{STG}\right),\forall m\in M \end{array}$$ (6)

Besides, the GP network updating rule is based on a reinforcement learning rule, as follows:

$$\begin{array}{c} \Delta w^{GP}=\beta \left(Y^{STR}-w^{GP}\right).\delta_{1} \end{array}$$ (7)

where *δ*_1_ = 1 if reinforcement, and 0 otherwise.

$$\begin{array}{c} Y^{GP}\left(t+1\right)=Y^{GP}\left(t\right)+noise.\delta_{\Delta E} \end{array}$$ (8)

where *δ*_Δ*E*_ = 1 if Δ*E* > 0, and 0 otherwise.

There are no inhibitory weights or neurons in the model, which is in contradiction with what is found in the Striatum. However, our framework is in line with the general assumptions of the cortico-basal loop functioning presented in [51, 71] in which the action of the reinforcement signal *δ*_Δ*E*_ acts as an inhibitory/learning signal. Our model of the basal ganglia has also some similarity with the Graybiel model in [54] in which the GP ‘expert systems’ are noisy generative models and are inhibited/modulated by a signal only during learning, when errors occur.

### Experimental setup

The aim of our experiments is to study the vocal learning and acoustic matching during self-supervised learning from the listening of one speaker or from several. The experimental setup for Experiment 1 in section consists of a small audio dataset of 2 minutes length of a native French woman speaker repeating five sentences three times. The audio .wav file is translated into MFCC vectors (dimension 12) sampled at 25ms each and tested either with a stride of 10ms or with no stride. A stride is the temporal shift between two samples. Typically, if we have one sound sample between [0, 25ms] then the next sample will be between [10ms, 35ms]. A stride of 25ms guaranties that there is no overlapping across samples. The whole sequence represents 14.000 MFCC vectors for the case with strides and 10.000 MFCC vectors for the case with no strides.

The numbers of Striatal and GP units are chosen so that they correspond to the number of MFCC vectors, which means 14000 units (or 10.000 units without strides) for each layer. We do so in order to test the reliability of our architecture to retrieve input data with an orthogonal representation. The compression rate is, however, low (1:1). We organize the MFCC vectors only depending on the temporal order of appearance in the Wav file.

In contrast, Experiment 2 in section will use a bigger audio dataset of 27 minutes length from six native French speakers, the same speaker as in Experiment 1 plus two other women and three men, repeating the same sentences as in the previous experiment. The audio .wav file is translated into MFCC vectors (dimension 12) sampled at 25ms each, which corresponds to 140.000 MFCC vectors for the case with 10ms stride. The numbers of Striatal and GP units are kept the same as for the first experiment (14.000 units), which means that the size for the BG layers is now ten times lower than the total number of MFCC to be retrieved in the sequence. The compression rate this time is high (1:10). This second experiment will serve to test the generalization capabilities of our architecture and its robustness to high variabilities with respect to the inputs, replicating the correspondence problem.

The sentences used in the audio database were selected because they cover all the syllables in French. Each period takes 10 minutes on a conventional laptop for the supervised method. The stabilization is done depending on the global error and we can decide below a certain threshold or we can choose a maximum number of iteration to stop the learn stage. For the unsupervised one, it can take much longer, 30 minutes to one hour to stabilize the dynamics below a certain error level. In our computation, we let the system stabilizes itself for a maximum of ten periods independently to a particular threshold level. We provide a link to .wav files samples and results as well as a link to source code at https://git.cyu.fr/apitti/inferno.

## Results

### Experiment 1—Self-supervised vocal learning of audio primitives

In section, we make the Primary Auditory Cortex (PAC), STG and Striatum layers learn in an unsupervised manner so that the three structures self-organize to sparse distributions using Hebb’s law for the PAC and the Striatum whereas the STG learns the temporal dependencies across time using the STDP learning mechanism; the direction of the information flow is PAC→STG→STR. In section, the GP layer learns audio primitives (the MFCC vectors) through free-energy optimization; the direction of the information flow is then STR→GP→PAC→STG→STR. We study the two cases where we leave the system unsupervised (self-organized regime) and where we control its dynamics (forced regime), resp. section and. The self-organized mode is done through a winner-takes-all, which means that the highest STR unit activity is the one selected. In the supervised mode, the PFC provides the desired STR unit to be selected. We analyze the performance of the Inferno architecture in section.

#### Striatal categorization of STG states

**Fig 4:**
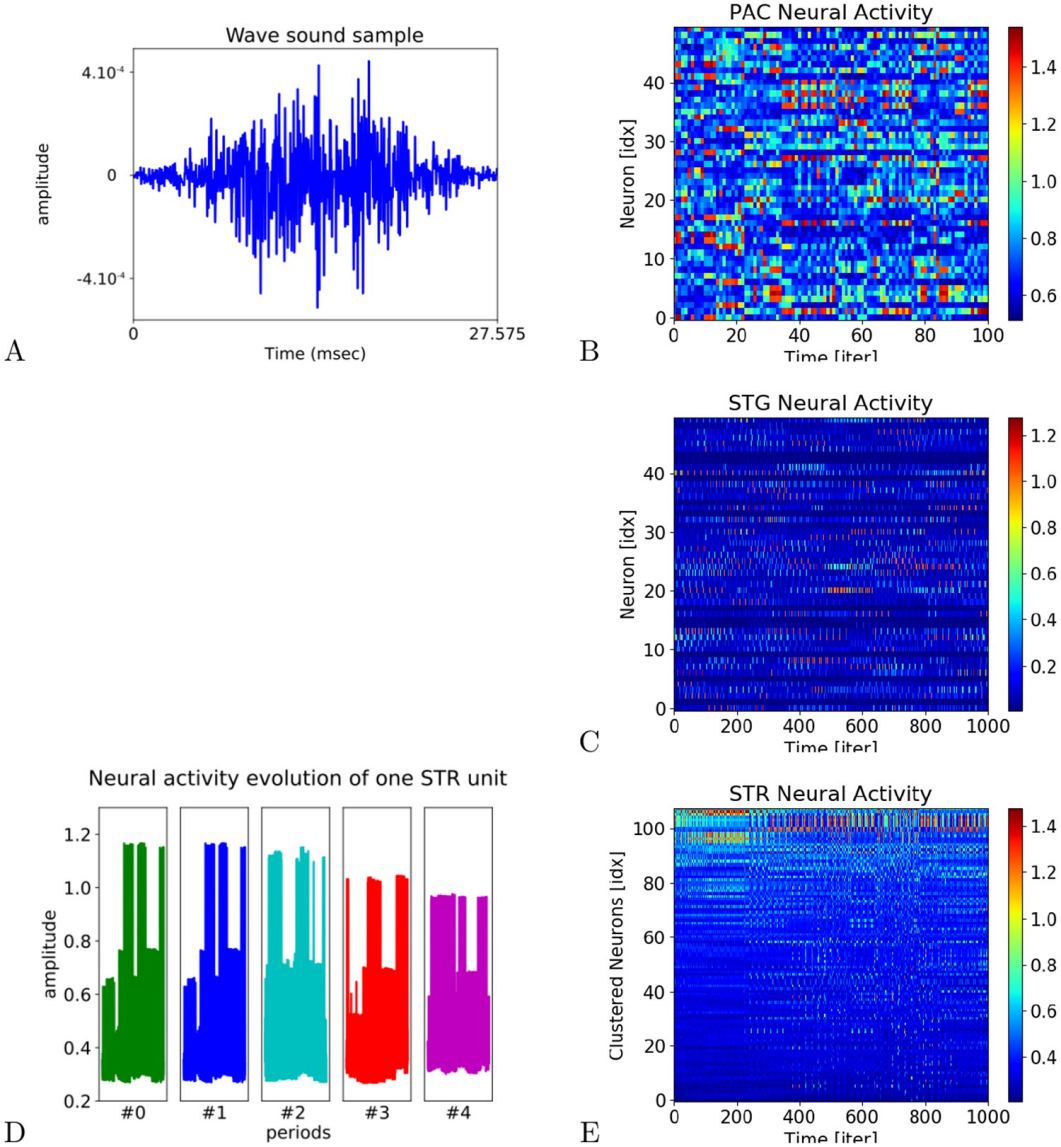
Dynamics of different structures during and after the learning stage. In A and B, waveform sample that the PAC layer categorizes in the form of MFCC vectors in a higher representation. In C, this information is passed to the STG layer that integrates over time (20 iterations) the incoming information. In D, evolution of the neural activity of one STR unit at different learning stages. In E, the final layer, the STR, categorizes for a second time the filtered information in a bigger neural population.

In order to understand the behavior of the system during the learning stage, we display the raster plots of the different dynamics for the PAC, STG and Striatum layers for 1000 iterations respectively in Fig 4B, 4C and 4E. The corresponding waveform sample is presented in Fig 4A and the evolution of one STR neuron activity is also presented at different learning periods in Fig 4D. While the PAC first receives the MFCC vectors at each iteration in Fig 4B, the STG integrates the different dynamics with a temporal horizon of 20 iterations, see Fig 4C. Then, a third layer, the Striatum (the STR network), categorizes the current state of the STG network in a higher dimension. A clustered version of its dynamics is displayed in Fig 4E to visualize better the neurons dynamics and the amplitude evolution of one neuron is presented over several learning stages to describe it in Fig 4D. We justify the need to have a Striatum network of dimension as large as the audio database in order to separate orthogonally the MFCC vectors.

So far, the learning stage is feed-forward from PAC→STG→STR and the categorization is done in an unsupervised manner. The plasticity coefficient added to the learning mechanism of the Striatal units in Eq 7 avoids any catastrophic forgetting after updating the weights several time, see Fig 4D. Over time, the dynamics of the STR network are less noisy, slightly diminish and stabilize demonstrating that a learning process is at work, as showed in Fig 4D.

#### CX-BG Iterative free-energy exploration-optimization

Once several periods are done over the complete audio sequence, the neurons stabilize to certain representations. It is possible then to perform an active exploration stage in the other direction—which means STR→GP→PAC→STG→STR for retrieving the corresponding audio entries in GP through reinforcement learning.

This stage corresponds to a motor babbling in which the audio inputs are generated in GP and evaluated after a delay in STR. The prediction error in STR is used to drive the dynamics in GP using free-energy and to control the PAC layer and STG dynamics via an iterative optimization process. Over time, each audio vector is reinforced for each GP-Striatal pair whenever the GP auditory pattern makes its corresponding Striatal unit fire. The audio pattern converges to an optimal MFCC vector for which the Striatal unit was the most active. As proposed by several neuroscientists, the GP layer may control indirectly the Striatal layer through the cortical dynamics [34, 54, 55]. The prediction error may drive the amount of noise within the system and the ratio between exploration and exploitation. This scheme corresponds to a predictive coding mechanism, which can solve the temporal credit assignment problem between causes (in GP) and delayed effects (in STG) as we found in [36].

**Fig 5:**
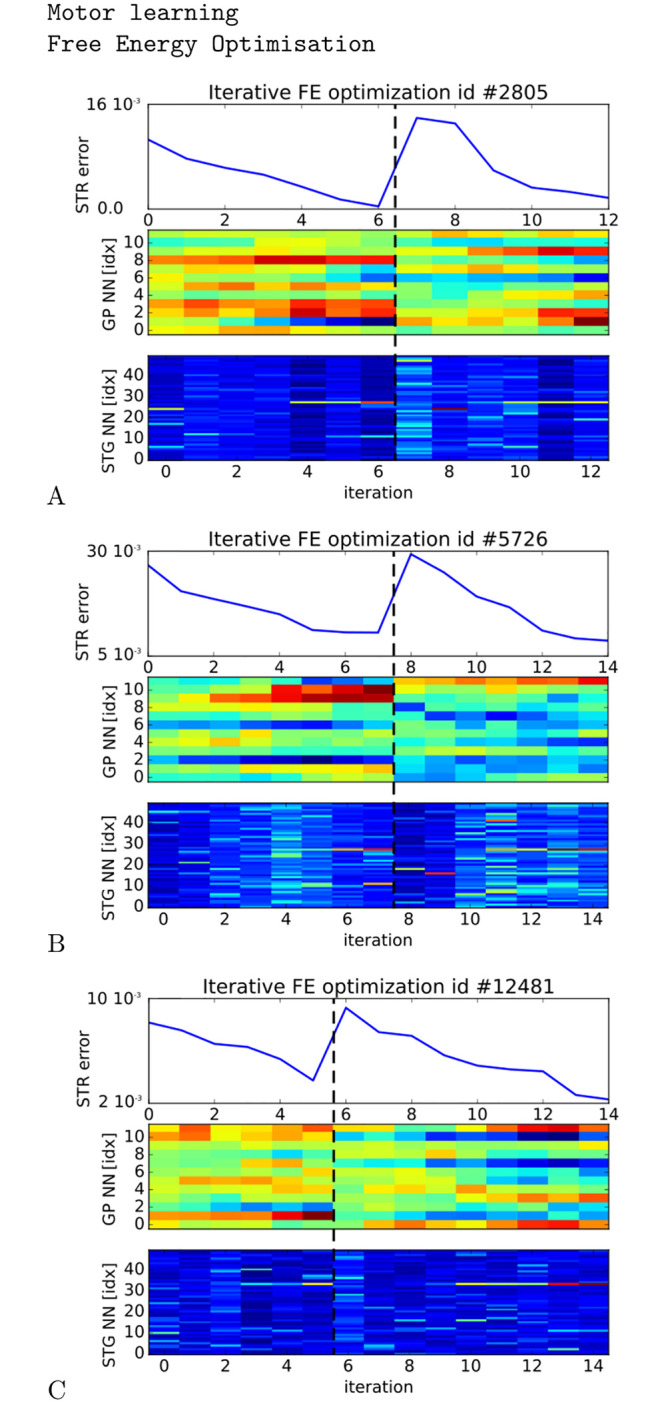
Free-energy optimization. A-C, error minimization of three Striatal units (top chart) using noise to retrieve GP vectors (retrieved MFCC vectors) for which the Striatal units fire maximally (middle chart). The STG units display different spike trains for which a solution is found (bottom charts). The dashed lines correspond to a reset of the GP dynamics (reset of the optimal MFCC vector) in order to show that the minimization process is always present and that different solutions can be retrieved dynamically.

We display in Fig 5 three examples of retrieved GP dynamics (middle chart) for which the prediction error in Striatum is diminished over time (top chart) with respect to the spatio-temporal patterns of the STG layer (bottom chart). The dashed line corresponds to a reset performed on the GP dynamics in order to observe dynamically the error minimization mechanism at work. The three samples correspond to the optimization process for three different Striatal units and for three GP vectors. During the free-energy descent gradient, each GP vector converges to one audio pattern for which the STG activity is the most recognized by the corresponding Striatal unit. As showed in the graphs, the optimization process does not necessarily converge to the same minima after the reset done on the GP vector but can be stacked to another one. This means that different patterns of activity in the GP layer can influence the activity in the STG layer in a similar way. Therefore, the categorization carried out in STR is not perfectly orthogonal (sparse) and different solutions coexist to retrieve the STG spatio-temporal dynamics.

**Fig 6:**
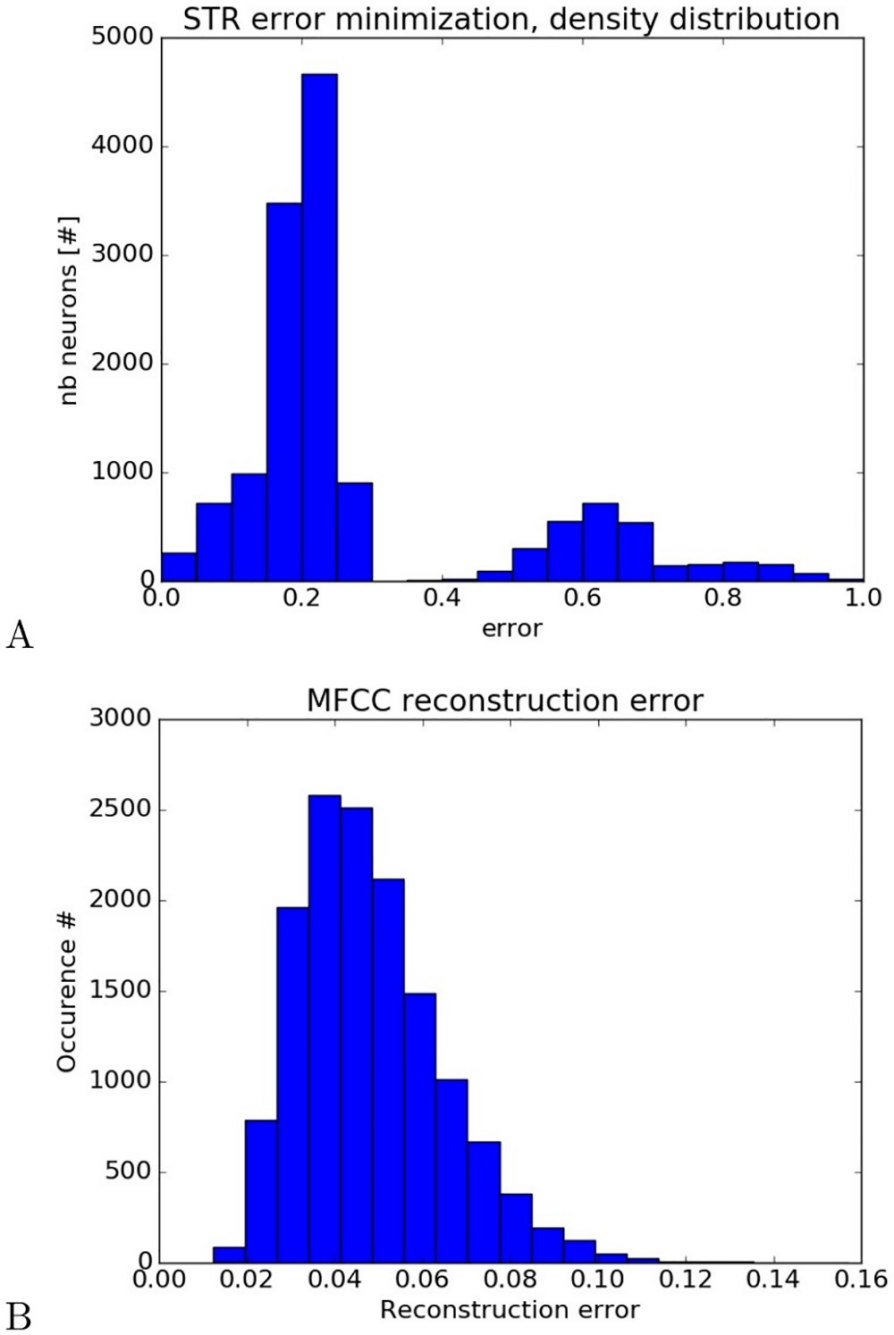
Reconstruction analysis after free-energy optimization. In a), density probability distribution of the Striatal units with respect to their prediction error level. In b), density probability distribution of reconstruction error of MFCC vectors by the GP layer. For most of the neurons within the STR layer, the optimization process makes it possible to construct MFCC vectors close to the real ones from the audio database. The error reconstruction follows a central field distribution centered at 0.05 and standard deviation ± 0.05.

We analyze in Fig 6 the learning performance of the free-energy optimization stage on the STR dynamics. Fig 6A presents the density distribution of the prediction error minimization for all the Striatal units and Fig 6B presents the reconstruction error in the GP units with respect to the MFCC vectors. In Fig 6A, the prediction error is computed as the difference between the maximal activity of neurons when triggered and their upper limit, which means that for an error equal to zero, the STR neuron is firing maximally whereas for an error equal to 1, the STR neuron is not firing at all. The result in this graph shows that for a majority of the STR units (80% of the population), the optimization process permits minimization the prediction error below a value of 0.3, which means that most of the GP neurons retrieved the optimal input vector that causes the STR to fire. Instead, for a small proportion of them (20% of the population), the error is above 0.4, which means that the optimization process was not effective. In this case, the INFERNO architecture did not find the relationship between auditory input and the striatal category.

In Fig 6B, the reconstruction error is computed as the Euclidean distance between the MFCC vectors presented in the audio database with the nearest GP vectors retrieved through free-energy optimization after normalization. The density probability distribution normalized between [0, 1] shows that the reconstruction process is good with an approximation error centered at 4%. The GP layer has found most of the MFCC vectors.

**Fig 7:**
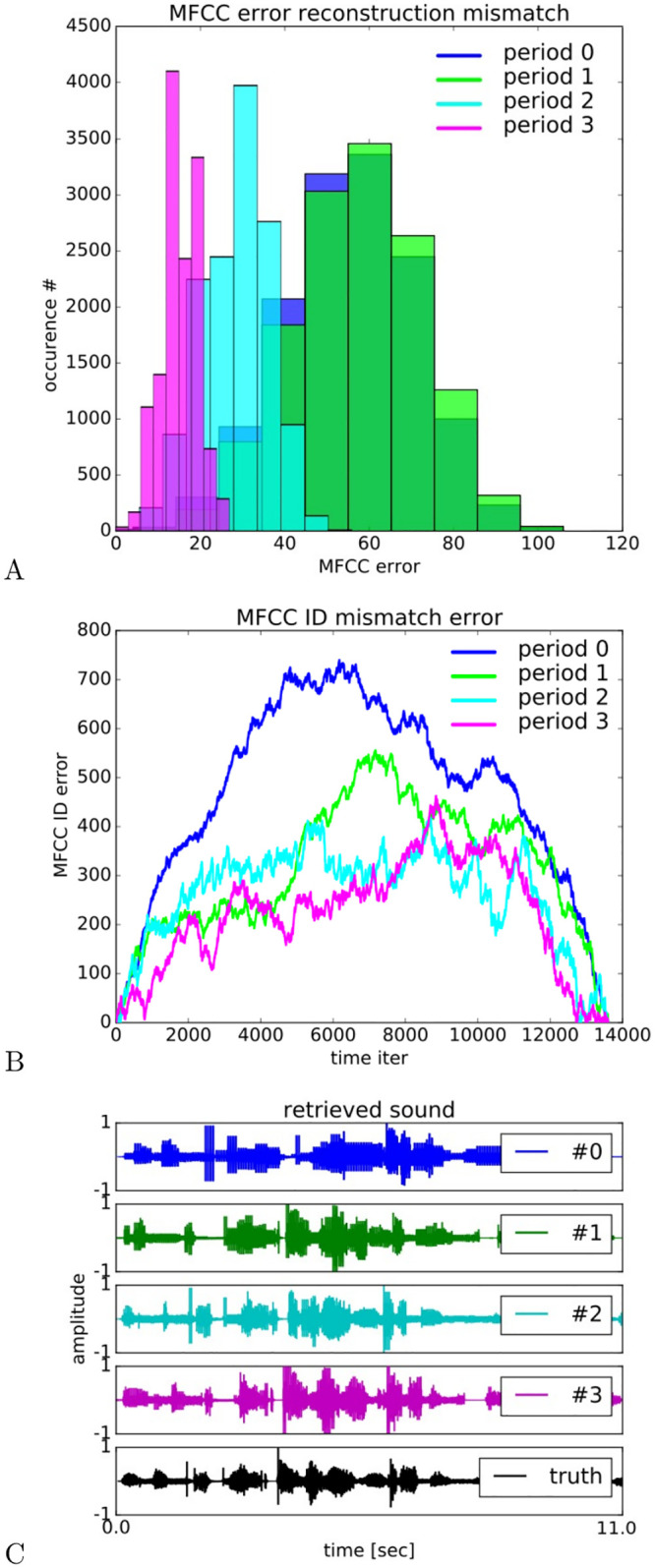
Performance analyzis after several exposures and reconstruction analysis of the audio signals. In a), Euclidean distance between the MFCCs retrieved and those from the audio database. In b), identity mismatch between the predicted MFCCs index and the correct one for the whole audio sequence. In c), waveform reconstruction for the four learning periods.

We present in Fig 7 further statistical analysis of the retrieved sound signals. In Fig 7A, we show a histogram for the MFCCs reconstruction error over 4 periods processing right across the audio sequence. The error is computed with the Euclidean distance between each GP vector with the nearest MFCCs from the audio samples. The error is not normalized between [0, 1] as in Fig 6B, the MFCCs vary between [0, 1200]. After each period, the error on each sample follows a distribution with lower error mean and narrower variance. The iterative optimization process goes from a 12% error to a 2% error on average on the samples. This shows the efficiency of the reinforcement learning stage in reconstructing the input dynamics.

A different curve is plotted in Fig 7B obtained from a Euclidean measure of the *identity* mismatch between the retrieved MFCC index and the correct one (ground truth) and displayed ordered in time within the sequence; therefore vectors with same index will have zero error. This measure should not be mistaken with the previous one as it computes the Euclidean distance between index of MFCCs and not between the MFCC vectors. The direct plot of the ‘MFCC error’ was rather difficult to read and we preferred this ‘meta’ distance to ease the comprehension. A low level indicates that the index of retrieved MFCC vector expected is near the real one and a high level indicates that the indices do not match. As similar to the previous figure, the error distribution diminishes gradually after each pass on the sequence. We can also observe that at the beginning and at the end of the sequence, the relative error is rather small corresponding to background noise when the person did not start speaking and when she ended up in advance.

When reconstituting the .wav file in Fig 7C from the retrieved MFCC vectors, we can observe a gradual refining of the audio waveform from the four periods with respect to the ground truth displayed at the bottom chart. The sequence is shown for 11 seconds although the global test was performed over two minutes length of the audio database.

After four exposures of the neural architecture to the audio sequence, the retrieved signals gradually converge to the correct waveform. At period #0, the waveform is very discrete with square-like pattern and the amplitude and the wavelength are not respected. Gradually, from period #1 to #3, we can observe a refinement of the waveform matching the ground truth curve. We provide the link of the different .wav files at https://git.cyu.fr/apitti/inferno.

#### Self-supervised learning

**Fig 8:**
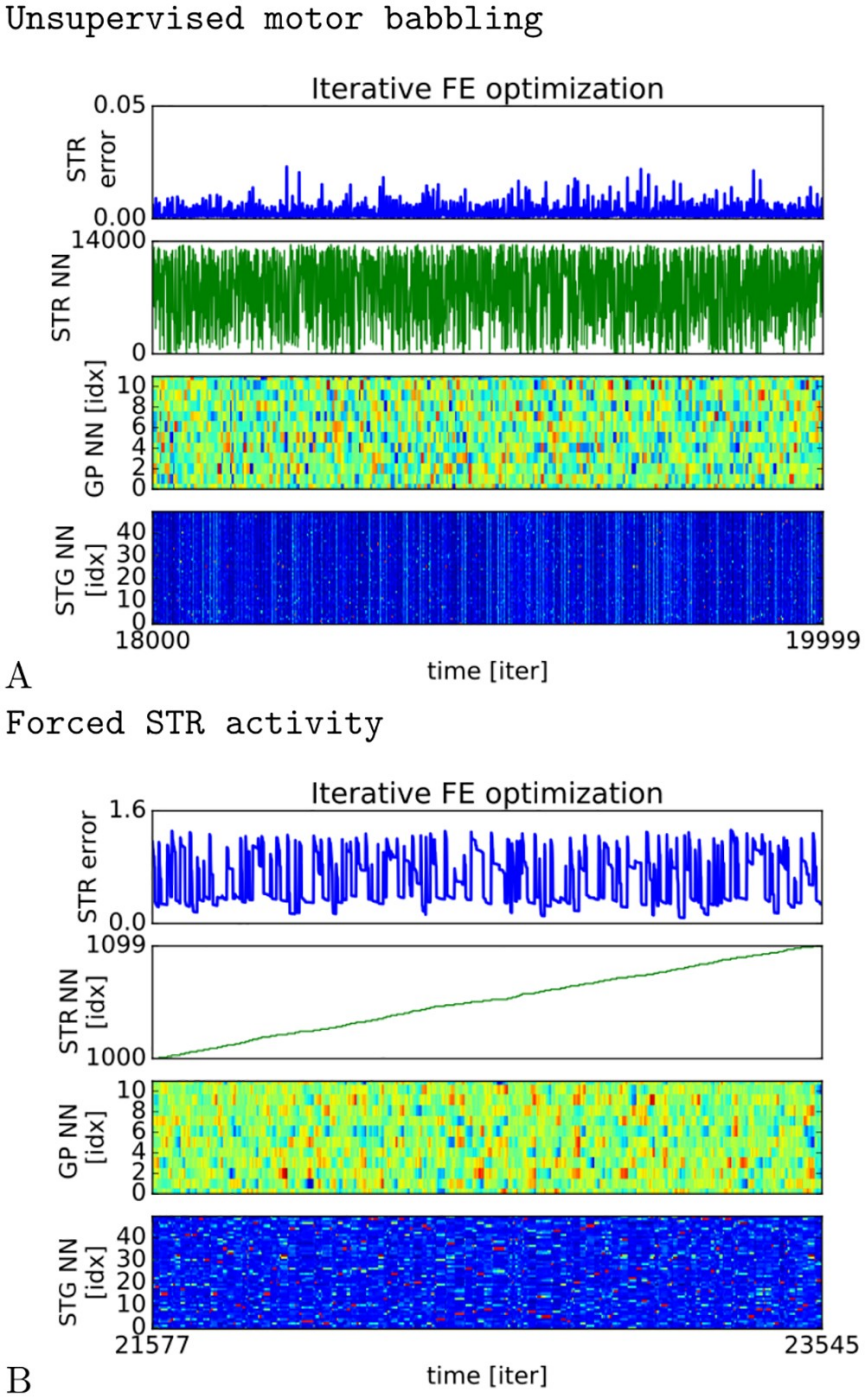
Self-supervised VS forced learning. We compare the two learning strategies resp. in A and B, in terms of convergence and dynamics. the self-supervising strategy might correspond to a babbling stage in which each audio unit is selected and tested at each cycle in a random fashion. Instead, the forcing strategy makes it possible to control the learning of each unit separately until convergence. In the supervised case (forced STR activity in B), the error is high for one specific STR unit in the beginning and then it is diminishing iteratively over time. We select one by one each STR unit until the error is diminishing to a certain threshold level during a limited amount of time, then the next neuron is selected to optimize the GP vector that optimally triggers the STG categories and the STR units. For the unsupervised case (unsupervised motor babbling in A), as at each iteration a different STR unit is selected because of internal noise, it is not clear to see such gradual decreasing of error for each unit.

The learning of the MFCCs does not need to be carried out in a specific order. It can be performed in an unsupervised manner by testing dynamically different sounds through cortico-basal recursion. This learning strategy may be seen as a motor babbling stage with random exploration. The resulting sequence is not necessarily coherent but at each iteration, the optimization process is at work to explore and improve the MFCC vectors found in GP. We present in Fig 8A the unsupervised learning of the GP units combined with the information processing done in the STR and STG layers for two thousand iterations. Below a certain error level (1st chart), the Striatal neurons have discharged maximally and another exploration cycle is engaged with the selection of a different Striatal unit (2nd chart). This second cycle will modify the dynamics in the GP (3rd chart), the PAC and the STG layer until (4th chart) maximization of the STR units. The recall is not instantaneous at the beginning of the cycle and several iterations are necessary to make the different layers converge. The process is similar to a greedy hill-climbing strategy although it is more visible in Fig 5.

#### Forced learning

As opposed to the unsupervised learning strategy presented previously, we can force the recall of the Striatal neurons in a specific serial order, see Fig 8B. This control is normally assured by another structure, the PFC, to retrieve an ordinal sequence. The PFC provides a teaching signal to STR. This signal consists in activating the STR unit we want to learn. As a consequence it bypasses the WTA stage in STR, and makes the winning neuron the desired STR unit. This forced recall is performed by the activation of the corresponding STR unit. This activation is done by the experimenter through the PFC (see section). This may also be done by the PFC alone (see [46]).

The error minimization stage takes a shorter time to converge to the optimum STG dynamics in comparison with the unsupervised learning strategy. However, the errors are higher than we might expect. Indeed, we used a maximum number of iterations per unit–, which permits to select directly the STR neuron we want to converge,– in order to do the supervised learning in one epoch only. Therefore the minimization process is more focused, effective and faster than the self-organized method–, which requires more epochs to converge,– in order to attain a similar error range. We could have a smaller error than 0.3 if we have added more iterations or chosen a lower threshold.

Comparing the two learning strategies, we found that the unsupervised learning with self-organization could achieve error minimization and control on the STG dynamics but the retrieving of longer sequences was not completely effective. These results are similar to what we found previously in [36]. Using unsupervised learning, the search space is not fully explored if the dimensionality is too large and the neural architecture can be trapped into local minima even if we use noise for descent gradient.

The learning stage can be very long and sub-optimal in comparison to the forcing method performed in a supervised manner. Over time, the supervised learning appeared more efficient at tutoring the INFERNO network by providing goals, when we force the activity level of one STR unit to a high state (see section) and minimize its error up to a certain threshold.

This is in line with the idea of intrinsic motivation [29, 30, 72, 73], that a goal-based approach plays a structuring role in comparison with a random-based approach, which will not take off if the dimension space is too large. Such a structuring role is perhaps played by the PFC and Hippocampus on the whole cortex during development [74]. The PFC and Hippocampus may play a structuring role on the whole cortex during a developmental stage [46, 75], while a model-free RL system alone is not enough for it.

#### Retrieved MFCCs & audio primitives

**Fig 9:**
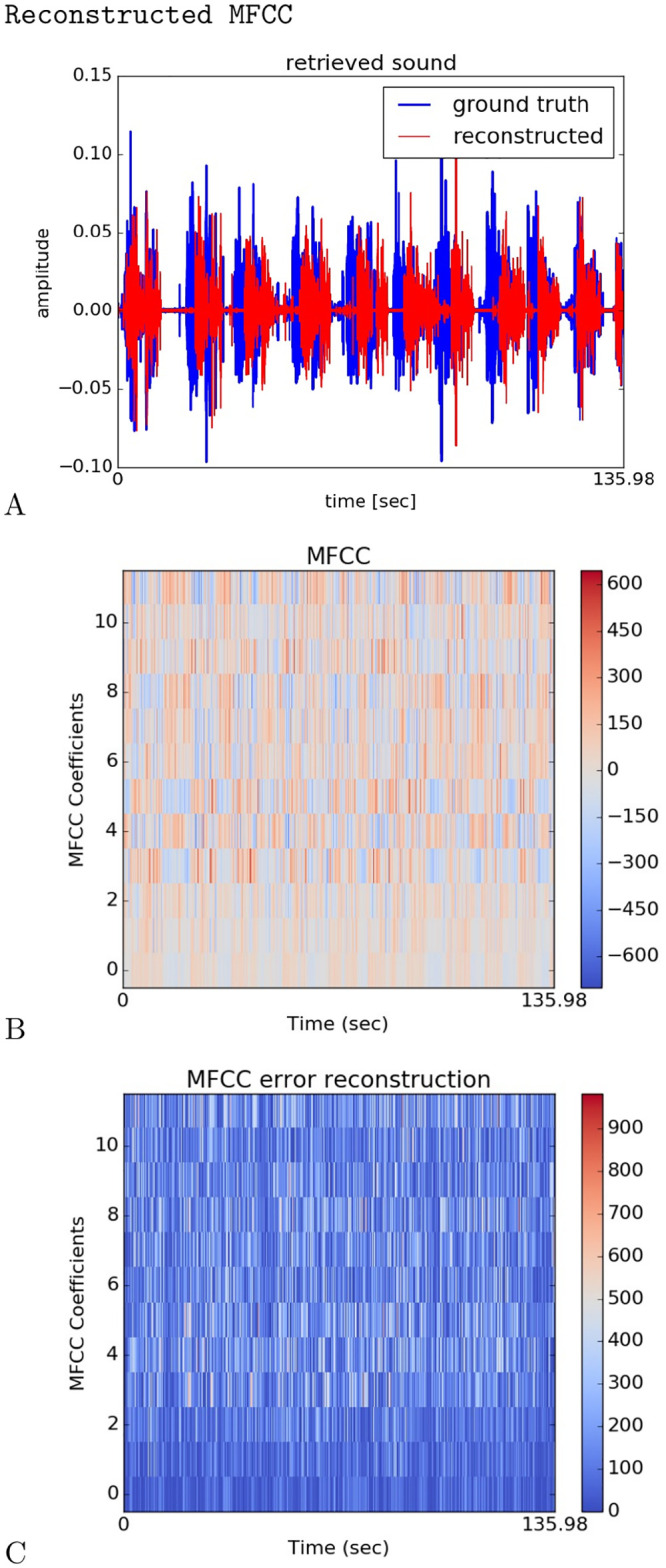
Reconstructed Waveform and MFCC comparison. In A, the original waveform is in blue and the reconstructed one is in red. In B, the reconstructed MFCC raster plot. In C, the raster plot of the MFCC error between the original sequence and the retrieved one.

We display in Fig 9A the reconstructed .wav signal (in red) with respect to the real signal (blue) (2 minutes length) from the MFCC retrieved in GP and realigned in the correct order, Fig 9B. The MFCC coefficient errors between the real signal and the one reconstructed are displayed in Fig 9C.

We can observe that the overall waveform of the sound signal is correctly reconstructed although some errors and some delays are visible and audible. The errors could likely be reduced with longer time for convergence, but we did not test this hypothesis in this experiment. The MFCC coefficient errors in Fig 9C show that the error is larger for the high MFCC coefficients (high pitch) than for the small MFCC coefficients (low pitch). As the smaller coefficients correspond to low frequencies, it makes sense that the important part of the signal, which is in the high frequencies, is harder to retrieve.

### Experiment 2—Correspondence matching with several speakers

In this section, we present the experiment carried out on a larger audio database with an architecture of the same size as in the previous section, which means with 14.000 STR units. The audio database of 27 minutes (140.000 MFCCs) is more difficult as it consists of sentences pronounced by six different speakers with equal numbers of each gender. As expressed in section, the sentences used here were selected because they cover all the syllables in French.

As the ratio between STR units and MFCC to be encoded is now 1:10, we here investigate the generalization and inference capabilities of the network during acoustic matching with unheared voices, known as the correspondence problem. This experiment reproduces some of the conditions faced by babies during acoustic matching when the audio repertoire learned is small and the heared voices are mostly unfamiliar and novel.

As we do not have access to the ground truth classes in the MFCC audio dataset, we cannot compute the basic classification analysis with recall and precision. Instead, we will analyze the performances in term of similarity between the original MFCC sound vector and the generated ones by the Inferno network. This measure is similar to the ABX distance proposed in [76, 77] for unlabeled audio database, and we will present it later.

The questions we would like to ask are: How well the motor and sound repertoires match novel voices? How robust is the categorization of unheared MFCC vectors in the STR (perceptual) layer and how similar is the reconstructed GP (motor) repertoire to the ground truth MFCC vectors? Differently said, how well what the network pronounces match what it listens?

**Fig 10:**
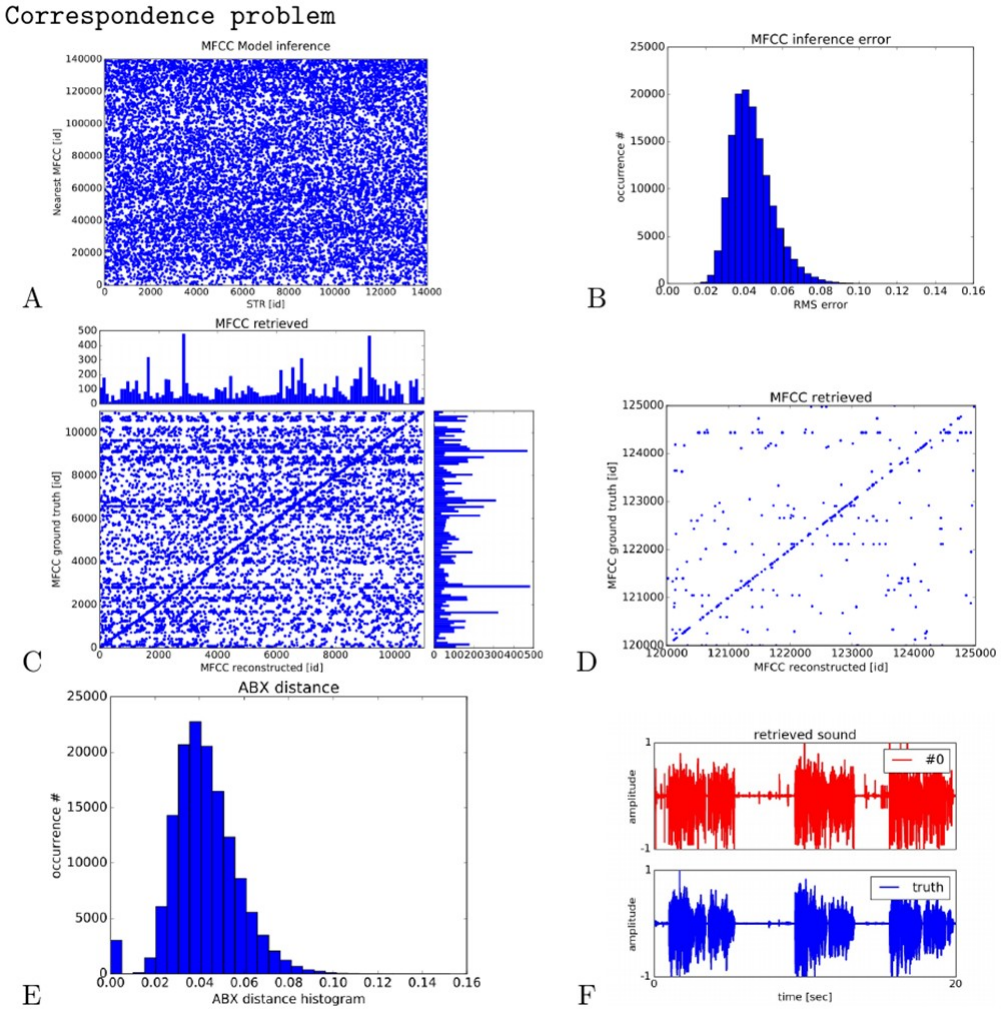
Analysis of STR reconstruction and MFCC mapping during acoustic matching with different speakers. In A, the correspondence matrix between STR units X and MFCCs vector A within the audio database of unheared voices. In B, the Euclidean distance between the MFCC vectors of the predicted STR units X with the ground truth MFCC vectors A within the audio database. In C the correspondence matrix between the ground truth MFCC vectors A and the nearest ones B from the reconstructed vectors X selected in STR, based on the correspondence matrix in A; plotted for the first 10.000 MFCC vectors. In D, a zoom in the correspondence matrix for 5000 units within the interval range [120.000; 125.000]. The diagonal indicates the good matching between what perceives the Inferno network and what it can pronounce, even from unheared MFCC samples during the learning stage. In E, the ABX distance histogram proposed by [76, 77] computed from the Euclidean distance between the A and B vectors retrieved previously. In F, an example of a retrieved waveform is provided from an unheared sound sequence after the learning stage.

We present in Fig 10A–10F different analysis carried out after the learning stage, resp. in a) the correspondence matrix between the retrieved indices of the STR units that match those of the ground truth MFCC vectors present within the audio database, in b) the Euclidean distance between the ground truth MFCC vectors present within the audio database and the retrieved MFCC vectors of the STR units, in c) the correspondence matrix between the ground truth MFCC vectors and the nearest MFCC vector also within the audio dataset which matches the closest the one predicted by the STR unit in a). A zoom in this MFCC correspondence matrix is plotted in Fig 10D and a histogram of the ABX distance computed from the correspondence matrix is presented in Fig 10E. Fig 10F displays a sample of the retrieved waveform.

The graph plotted in Fig 10A corresponds to the mapping between the STR units that match the closest the MFCC vectors in the audio database. The Euclidean distance is computed from the GP vectors retrieved in order to measure the correspondence between the MFCC and STR indices. Each MFCC within the audio database is predicted by only one STR unit. Conversely, each STR unit can code for several MFCC vectors. This shows the generalization capabilities of certain STR units within the network. For instance, we found that certain STR units cluster more than 100 MFCC vectors whereas others do not cluster any MFCC of the unheared database. Besides, we plot the Euclidean distance in Fig 10B to show the good generalization of the algorithm with a reconstruction error of 5%.

Using this mapping, it is possible to construct in Fig 10C and in 10D a correspondence matrix between the MFCC vectors A and B found in the audio database where A represents the ground truth vector and B the closest MFCC vector to the vector X generated by the STR units. The Euclidean distance between the A and B vectors computes then the ABX score proposed by [76] and plotted in Fig 10E.

The graphes in Fig 10C and in Fig 10D are constructed as follows. At first, we select the most probable STR unit X found in Fig 10A for each ground truth MFCC vector A. In second, we compare the MFCC vector generated with all the MFCC vectors in the audio database and select the nearest index B. We plot in c) a small portion of the database of the first 10.000 MFCC units out of 140.000 MFCCs units and in Fig 10D between the interval [120.000; 125.000] for a better visualization. A one-to-one correspondence between vectors A and B –, which means that they have the same index,– indicates a good generalization and a good matching by the Inferno network. Conversely, items of different index indicate the redundancy within the audio dataset and a mismatch with the Inferno network’s prediction, which is something expected within the correspondence problem task.

For instance, the diagonal indicates that the mapping is bijective and that the network has retrieved some perfect matching between MFCC vectors B closest to the STR units X and the MFCC vectors A in the audio database: there is a good mapping between what can perceive and what can “pronounce” the Inferno network. We found 3702 matches between the A and B vectors out of 14.000 MFCC vectors X; which corresponds to a similarity score and a perfect matching for 26% of the items in the new database with those of the original database. The horizontal stripes indicate the redundancy within the large audio database, as well as some classification errors by the network. The zoom in the mapping plotted in Fig 10D shows that a linear correlation is performed and that the noise due to classification errors is however not so large. The ABX distance histogram in Fig 10D shows an average error of 4% by the Inferno network with peaks at zero error, which is in average with other predictors’ performances but on different unlabeled audio databases [76, 77].

These results describe how the network performs on a large audio dataset when facing the correspondence problem, the discrepancy indicates that the number of vectors to be retrieved is high in comparison to the number of units within the network. However, the *perceptual tuning* constructed by the network, using Kuhl’s expression [1], permits to be robust to the extrinsic noise generated by unfamiliar voices. This is confirmed by the rather low Euclidean distance plotted in Fig 10B and the ABX score in Fig 10E between the ground truth MFCC vector and the generated ones by the Inferno network. The reconstructed waveform in Fig 10F plotted in red in comparison with the real waveform plotted in blue is one illustration of this: although the wave envelope is mostly preserved, the sound details are degraded. This is how the Inferno network imposes a dimensionality reduction and has attempted to limit discrepancy and reconstruction errors when facing the correspondence problem [1].

## Discussion

We have applied the neural architecture INFERNO to the retrieving of audio primitives by evaluating prediction errors. This neural architecture is based on free-energy minimization using recurrent spiking neural networks that model broadly the CX-BG loop, see [36].

In this paper, we have shown its efficiency in the challenging task of audio primitive generation and recognition during vocal learning and acoustic matching. The BG network rapidly explores and retrieves MFCC sound vectors by testing them stochastically through the CX layer. The more the striatal units recognize and predict the CX output, the stronger is the reinforcement of the link with the discovered GP units. At the end of this minimization process, the GP layer constitutes a sound repertoire of MFCCs. We however acknowledge that our implementation does not propose a strict plausible model of the Striatum. In a more biologically realistic version of it, inhibitory neurons should have been modeled to force the striatal control on the non-desired GP units.

The INFERNO network has two features, namely generalization and robustness to temporal delays. On the one hand, the number of units in the Striatum layer imposes a dimensionality reduction depending on the number of sound primitives to be learned (e.g., the number of MFCC vectors). On the other hand, the temporal chains formed in the CX layer makes it possible to solve the temporal credit assignment problem and to link causes and effects thanks to STDP.

In the first experiments in section we have designed the network with the same number of STR units as there are of MFCCs to be retrieved (14.000 units) in order to have an orthogonal representation with few overlapping items. These experiments were necessary to assess the robustness of the network particularly in high dimensions.

Although we have shown that the CX-BG network was capable of retrieving audio primitives in a self-organized manner, its exploration phase takes longer than in a supervised manner. The exploration of the audio primitives in a self-organized manner is similar to a motor babbling, which tests different sounds until convergence to the correct ones is achieved. In comparison to [36], the precise recovery of the temporal sequence was not possible due to the redundancy within the sound repertoire in GP with too many similar MFCC vectors. Conversely, it is acknowledged that the Basal Ganglia possess also a limited number of motor primitives. This result makes sense as we reconstruct audio MFCC vectors in the GP layer and not motor primitives, which possess lower dimensionality, as we should have with a vocal robot, a model of the articulatory system or with a vocoder. Despite the dimensionality problem, the BG-CX loop is known to encode conditioning responses and its role is not devolved to the control of the precise serial recall of sequences. Instead, the PFC is known to perform such executive control on the cortico-basal ganglia system to realize a precise control of temporal sequences. This second PFC-BG system is presented in the complementary article in [46].

In the second experiment in section, we performed the acoustic matching with several speakers constituting an audio sequence ten times longer than the previous ones (27 minutes .wav and 140.000 MFCC) in order to assess the generalization capabilities of INFERNO to higher dimensions with a limited number of sound primitives. For this purpose, we intentionally kept the number of sound primitives the same as in the first experiment (14.000 units) to investigate the acoustic matching when interacting with different speakers. Although the reconstruction error was important in comparison to the results in the first experiment, the network was still able to generalize correctly to this larger temporal sequence. This underlies the capabilities of inference of the architecture despite the large variability found in the database.

These attributes for generalization and inference appear in line with what happens during development when facing the corresponding problem. For instance, infants appear to learn a dictionary of prototypical sounds and to know how to adjust different voices and, different contexts in their mother tongue [1]. One difficulty is to know how speech is decomposed into distinct units to be analyzed. At the end of the developmental stage, a large number of sounds will seem similar to infants although they are different; e.g., “r” and “l” in Japanese. This phenomenon, occurring from 6 months to 18 months, is known as perceptual categorization in which discriminating capabilities are narrowing. During this period, infants appear to organize a repertoire of prototypical sounds with which they can compare and infer any sound they think to be the closest as a sort of ‘perceptual magnet’ [78, 79]. This repertoire is either perceptual, motor, or sensorimotor and the decision-making seems to correspond Bayesian inference in speech [80–82]. The Inferno network present such attributes.

In our present research, the sound repertoire encoded is only perceivable as audio primitives as encoded in the GP layer in the form of MFCC vectors. In future research, we will use audio datasets found in the litterature to compare our results with other models, for instance audio datasets designed for unsupervised learning and development modeling from the Zero Resource Speech Challenge [77]. We are also thinking of using a vocoder with an audio speaker in place of the MFCCs in order to generate a real sound with a microphone to retrieve the sound information from another channel. That is, we think that having a robot that can speak and listen will help it learn by itself and from its social environment in a more ecological fashion through embodiment following a developmental process [5–7, 83]. In this line, we also envision extending our framework to visual information for audio-speech recognition [16, 17, 84].

## Conclusion

In this paper, we presented a systemic model of the cortico-basal system (CX- BG) based on free-energy optimization in order to learn sound primitives through vocal babbling. We used the architecture INFERNO to solve the causal problem consisting on retrieving the motor primitives (MFCC vectors) that cause desired perceptual states (coded sound vectors). In extended work, we will modify our system to implement action with articulatory motions and vocal tracks. In our comprehension of the free-energy optimization strategy proposed by Friston [32], free-energy optimization is similar to an adaptive reinforcement learning process carried out between two or more learning structures that attempt to minimize error prediction from each other by anticipatory control, surprise or coordination using a variational signal, the Free-Energy gradient. Therefore, it brings the adaptation and learning problem into the framework of optimal control and of predictive coding.

In our study, the cortico-basal circuits allowed to process and to organize the learning between the audio sensory map and the motor control. Our neural architecture INFERNO permitted to combine reinforcement learning and spiking neural networks for constructing a large audio repertoire of sound units in an autonomous manner, via a vocal babbling stage. The results show that the architecture is robust to noise and could adapt to new speakers, therefore solving the corresponding problem. For instance, the learning stage was performed with one unique speaker whereas the validation stage was performed on six different speakers.

In a complementary paper in [46], we have modeled a second network composed of the basal ganglia and of the prefrontal system (BG-PFC) in order to learn the temporal structure within audio sequences; i.e., the temporal order of the items within the sequences or its syntactic rules. This second network models the processing done in the Broca area for rule-based behaviours using a gating mechanism. It demonstrated computational advantages and better performances in comparison to the state of the art LSTM deep network [85] on a relatively small audio database with a large number of classes, a difficult task for deep networks.
